# CD200 in CNS tumor-induced immunosuppression: the role for CD200 pathway blockade in targeted immunotherapy

**DOI:** 10.1186/s40425-014-0046-9

**Published:** 2014-12-16

**Authors:** Christopher L Moertel, Junzhe Xia, Rebecca LaRue, Nate N Waldron, Brian M Andersen, Robert M Prins, Hideho Okada, Andrew M Donson, Nicholas K Foreman, Matthew A Hunt, Christopher A Pennell, Michael R Olin

**Affiliations:** Department of Pediatrics, hematology/oncology, University of Minnesota, Minneapolis, MN 55455 USA; Department of Neurosurgery, Hospital Number 1 of China Medical University, Shenyang, China; Department of Neurosurgery, UCLA Medical Center, Los Angeles, CA 90095 USA; Department of Neurosurgery, University of California San Francisco, San Francisco, CA 94158 USA; Department of Pediatrics, University of Colorado, Denver Anschutz Medical Center, Aurora, CO 80045 USA; Department of Neurosurgery, University of Minnesota, Minneapolis, MN 55455 USA; University of Minnesota, Lab Medicine and Pathology, Minneapolis, MN 55455 USA

**Keywords:** Checkpoint inhibitors, Immunotherapy, Immune suppression, Brain tumors

## Abstract

**Background:**

Immunological quiescence in the central nervous system (CNS) is a potential barrier to immune mediated anti-tumor response. One suppressive mechanism results from the interaction of parenchyma-derived CD200 and its receptor on myeloid cells. We suggest that CD200/CD200R interactions on myeloid cells expand the myeloid-derived suppressor cell (MDSC) population and that blocking tumor-derived CD200 will enhance the efficacy of immunotherapy.

**Methods:**

CD200 mRNA expression levels in human brain tumor tissue samples were measured by microarray. The amount of circulating CD200 protein in the sera of patients with brain tumors was determined by ELISA and, when corresponding peripheral blood samples were available, was correlated quantitatively with MDSCs. CD200-derived peptides were used as competitive inhibitors in a mouse model of glioblastoma immunotherapy.

**Results:**

CD200 mRNA levels were measured in human brain tumors, with different expression levels being noted among the sub groups of glioblastoma, medulloblastoma and ependymoma. Serum CD200 concentrations were highest in patients with glioblastoma and correlated significantly with MDSC expansion. Similarly, in vitro studies determined that GL261 cells significantly expanded a MDSC population. Interestingly, a CD200R antagonist inhibited the expansion of murine MDSCs in vitro and in vivo. Moreover, inclusion of CD200R antagonist peptide in glioma tumor lysate-derived vaccines slowed tumor growth and significantly enhanced survival.

**Conclusion:**

These data suggest that CNS-derived tumors can evade immune surveillance by engaging CD200. Because of the homology between mouse and human CD200, our data also suggest that blockade of CD200 binding to its receptor will enhance the efficacy of immune mediated anti-tumor strategies for brain tumors.

**Electronic supplementary material:**

The online version of this article (doi:10.1186/s40425-014-0046-9) contains supplementary material, which is available to authorized users.

## Background

Immune suppression occurs naturally via multiple mechanisms [1,2], serving an important role by resolving inflammation and returning the tissue microenvironment to homeostasis. There is clear evidence now that many tumors employ various immunosuppressive mechanisms to evade immune surveillance and to promote tumorigenesis [2,3]. The two most studied immune inhibitory receptors are cytotoxic T lymphocyte antigen 4 and programmed cell death protein 1, both of which are expressed on effector T-cells [4,5]. Monoclonal antibodies specific for these receptors extend survival in subsets of cancer patients, presumably by blocking ligand binding to these receptors and preventing inhibition of T-cell effector functions [5]. These striking clinical results suggest that brain tumor immunotherapy would benefit from the identification and subsequent blockade of immune inhibitory pathways in the central nervous system.

An immune inhibitory ligand/receptor pair that maintains immune quiescence in the CNS is CD200/CD200R [6]. CD200 (OX2) is a highly expressed membrane glycoprotein with a broad tissue distribution. In the CNS, CD200 is expressed predominantly by neurons [6,7], down-modulating the activation state of perivascular macrophages and microglia through CD200R [8]. Mice express multiple CD200R isoforms that exhibit tissue-restricted expression and heterogeneity of function [9-12]. Recent studies have reported the expression of CD200R1 on microglia, macrophages, dendritic cells and a subpopulation of T cells. Ligation with its ligand, CD200, negatively regulates immune responses through multiple mechanisms [13-15] including the activation of CD200R on MDSCs promoting tumorigenesis [14].

## Results

### CD200 is overexpressed by many brain tumors

To gain a better understanding of the role of CD200 in CNS tumors, we first examined CD200 mRNA expression on a variety of tissues. The data demonstrate an overall statistically significant increase in mRNA transcript levels in normal brain compared to non-brain tissues (p = 0.001) (Additional file 1: Figure S1A). In addition, we observed increased CD200 expression throughout the brain with the exception of the choroid plexus, which was statistically lower than the rest of the brain combined (p = 0.005). Post hoc analysis determined a significantly increased expression in breast, kidney, lung (p < 0.001) and pancreas (p < 0.0001). Thymus failed to reach statistical significance. In addition, post analysis revealed a statistically significant difference between choroid plexus and frontal and temporal lobes (p < 0.001 and p < 0.05 respectively). Cerebellum, hippocampus, lumbar spinal cord, medulla, midbrain, pons and occipital lobe failed to reach statistical significance (Additional file 1: Figure S1B).

**Figure 1:**
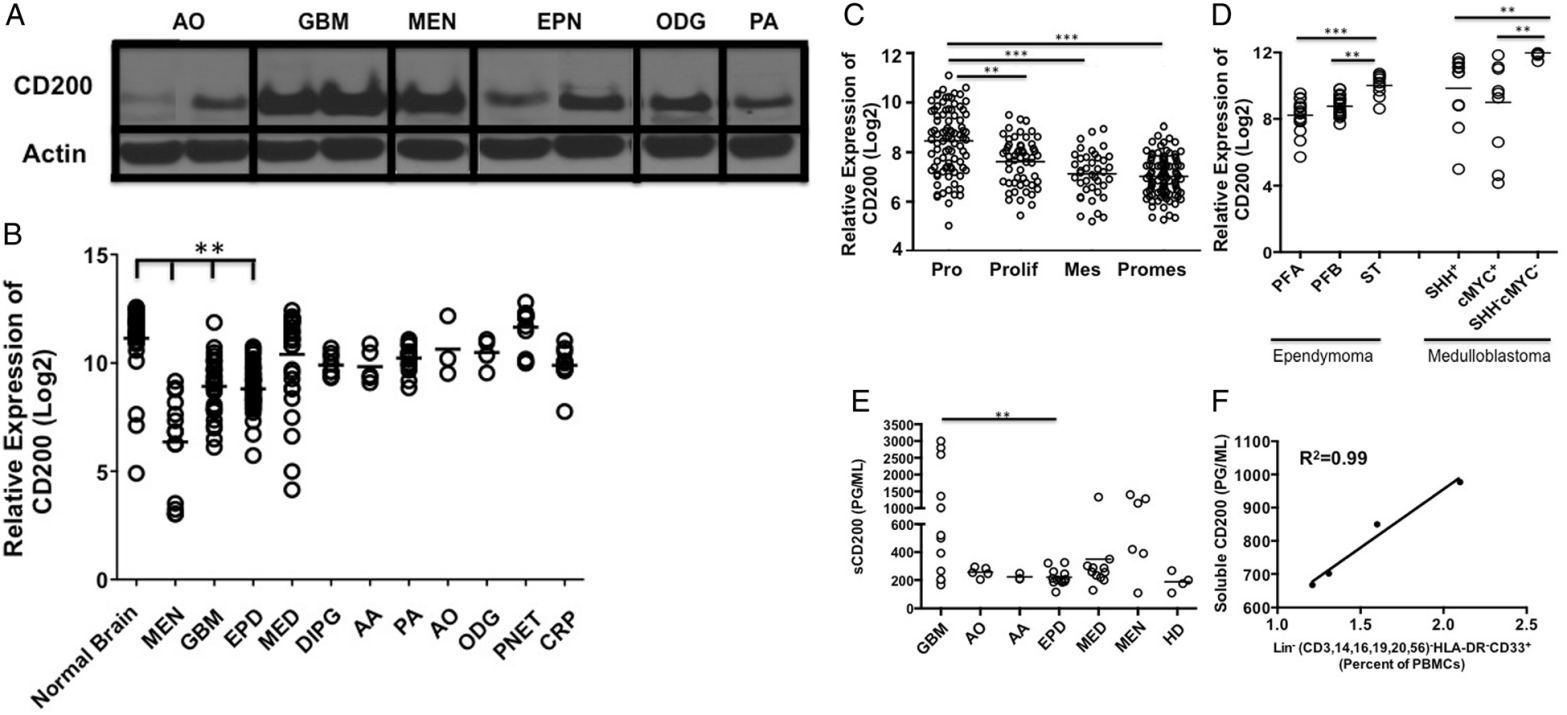
**Brain tumors express CD200. A**. Western analysis of CD200 on Anaplastic oligoastrocytoma; AO, Glioblastoma Multiforme; GBM, Meningioma; (MEN), Ependymoma; EPN, Oligodendroglioma; ODG and Pilocytic Astrocytoma; PA. **B**. mRNA CD200 expression levels in normal brain; (n = 30) and meningiomas; (MEN, n = 14) were compared to Glioblastoma Multiforme; (GBM, n = 31), Anaplastic Astrocytoma; (AA, n = 5), Pilocytic Astrocytoma; (PA, n = 17), Anaplastic Oligoastrocytoma; (AO, n = 3), Oligodendrogliomas; ODG, n = 4), Primitive Neuroectodermal; (PNET, n = 13), Medulloblastoma; (MED, n = 30), Ependymoma; (EPD, n = 45) and Craniopharyngioma; (CRP, n = 11). **C**. mRNA CD200 expression levels on Proneural; PRO Glioblastoma Multiforme subsets compared to Proliferative; Prolif, Mesenchymal; Mes and Promesenchymal; Promes. **D**. CD200 mRNA expression levels were compared between Ependymoma subsets Posterior Fossa A (PFA), Posterior Fossa B (PFB), Supratentorial (ST) and Medulloblastoma subsets Sonic Hedgehog positive (SHH^+^) compared to group 3 (cMYC^+^) and group 4 (Sonic Hedgehog negative/cMYC negative (SHH^-^cMYC^-^)) subsets. In addition, **E**. Serum concentrations of soluble CD200 were analyzed from patients bearing Anaplastic Oligoastrocytoma; AO, Anaplastic Astrocytoma; AA, Ependymoma; EPD, Medulloblastoma; MED, Meningioma; MEN and healthy donors; HD. **F**. Serum concentration of soluble CD200 from patients bearing glioblastoma multiforme was correlated to expansion of lineage negative MDSC population. Means are indicated, statistical significance was determined by one-way ANOVA, post hoc analysis by Dunn’s multiple comparison test, *p < 0.05, **p < 0.001. R^2^ was determined using linear regression.

Given its high expression on normal CNS tissue, we investigated the expression of CD200 on multiple central nervous system tumors. We found CD200 protein expressed on a variety of human brain tumor tissue samples by western analysis: anaplastic oligoastrocytoma (n = 1), GBM (n = 3), meningioma (n = 1), ependymoma (n = 2), oligodendrogliomas (n = 1) and pilocytic astrocytoma (n = 1)(Figure 1A). Given these initial findings, we next compared CD200 mRNA levels in a larger cohort of brain tumors relative to normal brain and meningioma (MEN), an extra-axial tumor arising in the lining of the CNS, not a true brain tumor. We observed a statistically lower expression (p < 0.0001) on meningioma (MEN), glioblastoma multiforme (GBM) and ependymoma (EPD) compared to normal brain. Meningiomas were significantly lower (p < 0.0001) than all other tumors.

Interestingly, further analysis of GBMs revealed an increased expression of CD200 in the proneural subtype compared to proliferative (p < 0.001), mesenchymal (p < 0.0001) and promesenchymal tumors subsets (p < 0.0001) (Figure 1C). We chose to look at promesenchymal gene expression based on studies by Nelson, et al. and Prins, et al. [16-18]. They reported a subset that featured both proliferative (classical) and mesenchymal groups, which was subsequently named promesenchymal. This was published in BMC Medical Genomics in 2008 and corroborated by other groups.

In addition to gliomas, we also observed a statistically significant increase in the supratentorial ependymoma subset compared to posterior fossa group A and posterior fossa group B (p = 0.0001 and p = 0.001 respectively). In addition, CD200 mRNA levels were significantly elevated in the group 4 (sonic hedgehog/cMYC negative) medulloblastoma subset compared to sonic hedgehog positive (SHH^+^) and group 3 (cMYC^+^) subsets (p = 0.001 and p = 0.001 respectively) (Figure 1D) [19,20].

CD200 is expressed on multiple tumor types [21,22]. Wong, et al. reported CD200 on cells from chronic lymphocytic leukemia patients [23], however, they went on to state that the soluble form of CD200 (sCD200) in the sera of patients is what correlated with poor patient outcomes [23]. Therefore, we analyzed sera from brain tumor patients for sCD200 levels. We observed a significant difference in sCD200 levels between patients with various brain tumor types by ANOVA (p = 0.001). Post analysis revealed a significant increase in sCD200 in patients with glioblastoma multiforme GBM compared to patients with ependymoma (p < 0.001) (Figure 1E). Three patients in the GBM group with the highest concentrations of sCD200 had aggressive recurrent disease. Furthermore, the highest concentration in the medulloblastoma group was from a patient in our clinical trial [24] who rapidly went off trial due to tumor progression. Interestingly, an ependymoma patient in the same trial had a statistically significant increase of sCD200 (p < 0.01 between weeks 4 and 12) as she went off trial due to recurrence (Additional file 2: Figure S2A). Together, our data support the reported correlation between elevated sCD200 and poor prognosis.

One potential mechanism by which CD200 induces an immune suppressive environment is through the expansion of MDSCs [10]. Therefore, we sought to correlate sCD200 in patients’ sera with the percentage of peripheral blood lineage negative MDSCs. The patients were all participants in a recent clinical trial designed to test the safety of our allogeneic vaccine [24] and included those with ependymoma (n = 1) and GBM (n = 3); correlations between sCD200 in the sera and MDSC frequencies were made just prior to vaccination, and at 4, 8, and 12 weeks post-vaccination [24]. There were significant correlations between sCD200 and expansion of MDSCs in the GBM patients (Figure 1F; R^2^ = 0.99) and ependymoma patient (Additional file 2: Figure S2 B; R^2^ = 0.97). These data suggest a direct correlation between sCD200 and MDSC expansion.

### CD200 pathway blockade reduces immune suppression induced by sCD200

**Figure 2:**
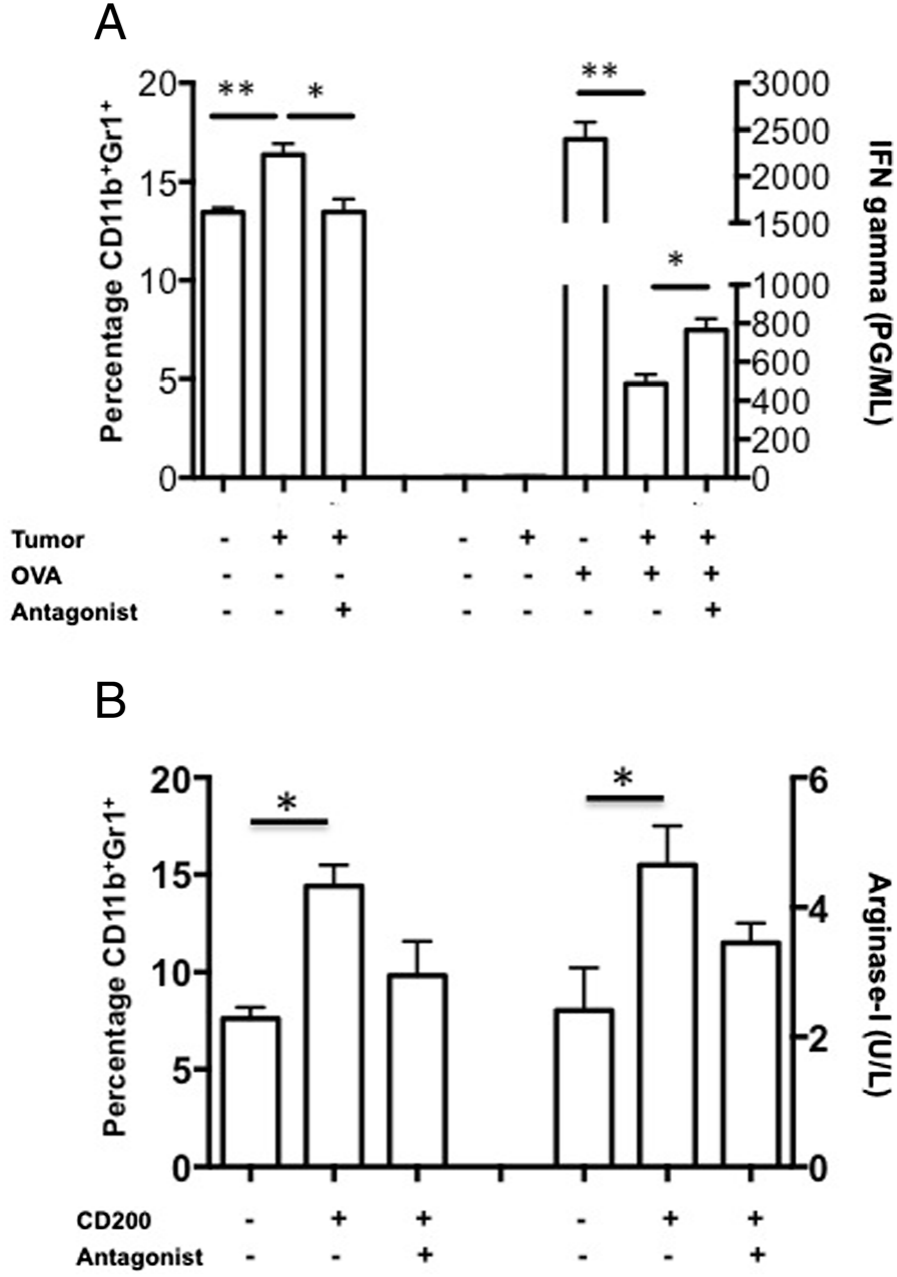
**CD200R antagonist blocks MDSC expansion and tumor suppressive effects. A**. Murine glioma GL261 was incubated with naïve splenocytes in a trans-well plate +/- CD200R antagonist and analyzed for MDSC expansion and cytokine production. **B**. Naïve splenocytes were pulsed with purified CD200 +/- CD200R antagonist and analyzed for MDSC expansion and arginase-I production. Error bars are ± SEM, statistical significance was determined using an unpaired T-test, *p < 0.05, **p < 0.001.

Based on the work of Gorczynski, et al. [9], we synthesized a CD200R antagonist peptide (A26059) to determine the ability of tumor secreted CD200 to block the expansion of MDSCs. The addition of GL261 glioma tumor cells induced a significant expansion of CD11b^+^Gr1^+^ MDSC (p < 0.01) and suppressed the ability of purified OT-I CD8 T-cells to respond to OVA (SIINFEKL) stimulation (p < 0.01) (Figure 2A). The addition of the CD200R antagonist blocked both the expansion of MDSCs as well as the production of IFN-γ (p < 0.01 and p < 0.01 respectively) (Figure 2A).

To validate that MDSC expansion is due to CD200, naïve splenocytes were pulsed with purified recombinant CD200 protein with and without the CD200R antagonist (A26059). These experiments show that CD200 alone significantly expanded the MDSC population (p < 0.01). Pre-incubating cells with the CD200R antagonist significantly blocked CD200-induced MDSC expansion (p < 0.01) (Figure 2B), and blocked arginase-I secretion (p < 0.01) (Figure 2C). There was a significant increase of MDSCs between treatment groups using the antagonist compared to a control antagonist (p < 0.001) (Additional file 3: Figure S3A).

### CD200R antagonist blocks tumor induced suppression resulting in an extension in survival glioma bearing mice

**Figure 3:**
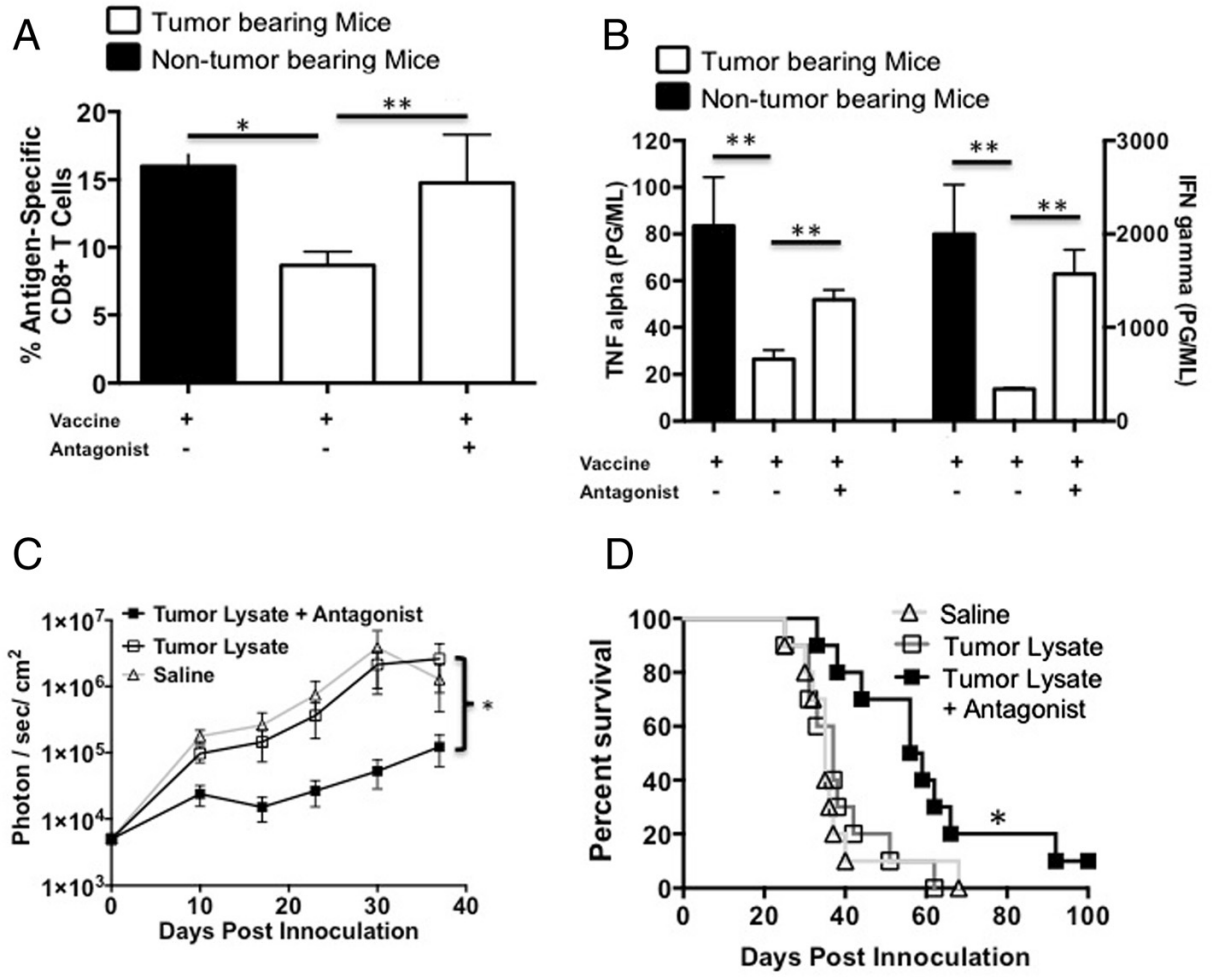
**CD200R antagonist blocks CD200 induced immune suppression enhancing survival.** **A** and **B**. Tumor bearing mice were vaccinated with OVA + Poly:ICLC +/- antagonist then analyzed for OVA-specific T cells and cytokine production following in vitro restimulation with OVA. Tumor bearing mice were vaccinated with saline (n = 10), tumor lysate + CpG (n = 10) or tumor lysate + CpG + antagonist 6059 (n = 10). **C**. Mice were imaged weekly for tumor growth and **(D)** followed for survival. Error bars are ± SEM, *p < 0.05, **p < 0.001 was determined by one-way ANOVA, post hoc analysis by Dunn’s multiple comparison test, log-rank analysis was used for survival. Error bars are ± SEM, statistical significance was determined using an unpaired T-test, *p < 0.05, ** p < 0.001.

We previously reported that vaccinating GL261 glioma bearing mice near the sentinel (cervical) lymph nodes failed to elicit an effective tumoricidal response [25]. In addition, we demonstrated, as described above, that glioma cells have the ability to enhance a MDSC population (Figure 2A). Therefore, we investigated the ability of our CD200R antagonist to block/reverse the *in vivo* suppressive effects of sCD200. Tumor bearing and non-tumor bearing mice were vaccinated in the back of the neck with OVA + Poly:ICLC to induce an antigen specific cellular immune response. The data presented in Figures 3 A and B show that the percentage of OVA specific SIINFEKL binding CD8^+^ T-cells (p < 0.01) as well as the ability to induce TNFα and IFNγ are significantly suppressed (p < 0.001 and p < 0.01 respectively) in OVA primed GL261 bearing mice (white bars) compared to non-tumor bearing mice (black bars). To investigate the potential role of CD200 in GL261 glioma induced immune suppression, we incorporated the CD200R antagonist 6059 into our vaccine inoculum. Tumor-bearing mice treated with the CD200 antagonist one day prior to and concurrently with OVA vaccine had increased numbers of SIINFEKL-specific CD8 T-cells compared to mice vaccinated without the antagonist (p < 0.01) (Figure 3A). Moreover, lymphocytes isolated from the cervical lymph nodes of mice vaccinated with the addition of the CD200R antagonist had significantly enhanced TNFα and IFNγ production (p < 0.01 and p < 0.001, respectively)(Figure 3B). These experiments suggest that CD200 plays a role in suppressing the immune responses in GL261 tumor bearing mice.

We next investigated whether the CD200R antagonist could enhance survival in our GL261 mouse model. Mice were given the CD200R antagonist 6059 one-day prior to and concomitantly with vaccination. We observed a statistically significant inhibition of tumor growth in mice vaccinated with antagonist compared to mice vaccinated with tumor lysates alone (p < 0.001) and mice that received saline only as a control (Figure 3C). The addition of the CD200R antagonist with the vaccine significantly slowed tumor growth (p < 0.01), resulting in enhanced survival benefit (p < 0.01) compared to other treatment groups (Figure 3C and D).

### Modified CD200R antagonists enhance survival in glioma and breast carcinoma models

**Figure 4:**
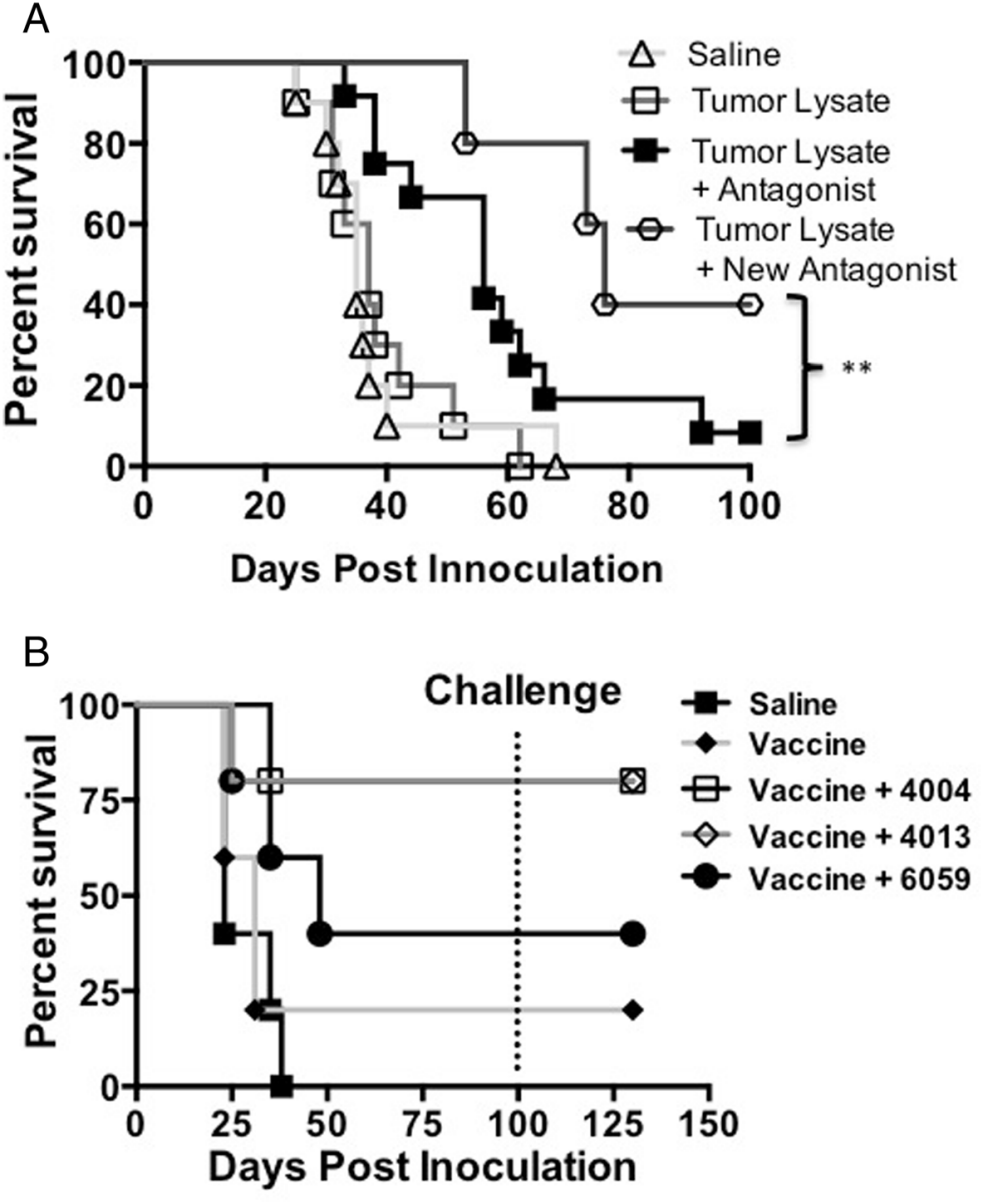
**Modified CD200 antagonists enhance survival. A**. EMT6 tumor bearing mice were vaccinated with tumor lysate, CpG +/- CD200 antagonist 4004, 4013 or 6059. **B**. New CD200R antagonist A12-6059 enhances survival. Log-rank analysis was used for survival.

Gorczynski reported that multiple regions of the CD200 act as antagonist, blocking the suppressive effects of CD200 [9]. Ongoing investigations of another CD200R antagonist demonstrates even greater survival (p < 0.001) (Figure 4A) compared to the 6059 in our GL261 glioma model. Subsequent experiments demonstrated that decreased tumor growth is due to the use of our new antagonist (A26059). Using control peptide failed to inhibit tumor growth (Additional file 3: Figure S3 B). However, differences between mice given a CD200R antagonist and the control antagonist failed to reach statistical significants.

To test if our antagonist was efficacious in a non-CNS tumor, we tested multiple CD200 antagonists on our breast carcinoma model. CD200R antagonists 4004, 4013 and 6059 showed enhanced survival benefit in an EMT6 breast carcinoma model (Figure 4B). Interestingly, CD200R antagonist 4013 and 4004 failed to confer survival in our glioma model (data not shown). All mice were tumor free by day 100 when they were challenged with 1 × 10^6^ EMT6 cells. Regardless of the CD200R antagonist used, all mice rejected challenge without further vaccinations. These experiments demonstrate the ability of small molecule peptides to mitigate the negative signals of the CD200/CD200R interaction.

## Discussion

Because of the myriad of ways tumors induce immunosuppression, it is unlikely that any monotherapy will be completely effective. Therapy using a combination of vaccine, adoptive cell therapy, checkpoint inhibitors or other immune-stimulating therapies will likely have better results. Several immunotherapeutic approaches, such as agents targeting the known immune checkpoints, have been developed and are under evaluation as cancer interventions [26-28]. We found increased expression of CD200 mRNA in multiple human brain tumors as well as increased soluble CD200 (sCD200) concentrations in the sera of patients as their tumors progressed. A CD200R antagonist peptide mitigated glioma-induced immune suppression in mice. The combination of this peptide and a glioma-derived vaccine significantly extended survival in glioma-bearing mice. Our data suggest that gliomas have co-opted CD200-mediated immune suppression to persist, and that blocking the CD200/CD200R immune checkpoint is a viable strategy for glioma therapy.

We believe that the soluble form of CD200 (sCD200) derived from tumors is carried to the cervical lymph nodes through the cerebral spinal fluid, inducing negative signals by placing severe limitations on the ability of the immune system to mount a tumoricidal response following vaccination. In contrast to non-CNS tissues, we detected high expression levels of CD200 throughout the brain with the exception of the choroid plexus (Additional file 1: Figure S1 A & B). It was not surprising that brain tumors, due to the origin of the cells, had high expression levels of CD200 (Figure 1B). However, compared to most CNS tumors, GBMs had lower expression of CD200. Therefore, we investigated data derived from the TCGA database. Expression of CD200 was down regulated in 2% of the RNAseq data (3 out of 153 tumors), 6% of the U133 Microarray data (33 out of 528 tumors) and 7% of the Agilent Microarray data (36 out of 500 tumors). Overall, altered expression of CD200 is detected in 6% of ~1200 GBM tumors. Altered CD200 expression in GBM tumors from the TCGA database (Grade IV) did not correlate with survival or relapse (Log-rank p-value not significant). Likewise, altered expression of CD200 in GBM tumors from the REMBRANDT database (grades II, III, IV) did not correlate with survival.

Interestingly, none of the altered CD200 samples from RNAseq or U133 Microarray data co-occurred with IDH1 mutations (~5% of GBM tumors), however, no significant association was calculated (in this case, by mutual exclusion). In the Agilent Microarray CD200 up-regulated samples, only one of the samples co-occurred with an IDH1 mutation and, again, no significant association was calculated (co-occurrence or mutual exclusivity). It is important to note these statistical results may be due to small sample size but the non-overlapping trend of altered CD200 expression and IDH1 mutations is interesting. No IDH2 mutations were detected in any of the GBM tumors analyzed for mRNA expression.

We have found that CD200 expression varies within different tumor types, and that specific tumor subsets also have varying expression levels of CD200. We saw a significant increase in CD200 expression levels in the proneural subset of GBM compared to mesenchymal GBM. This is interesting because the proneural subset is associated with minimal immune cell infiltrate, limiting immune-tumor cell interactions [18]. However, patients with the proneural subset of GBM have a more favorable prognosis than the mesenchymal subset, which is associated with high cell infiltrates. This may be due to the low accumulation of MDSCs or M2 type macrophages that permit tumor growth [29]. The same trend may be true for ependymoma and medulloblastoma subsets, posterior fossa A and group 3 (cMYC^+^) subgroups, respectively, which are more aggressive, have low CD200 expression and exhibit high immune infiltrates [19,30,31].

CD200 negatively regulates immune function through interactions with the CD200 receptor [10]. CD200/CD200R interactions result in the expansion of suppressive cell populations such as MDSC [32]. In our studies, we observed a positive correlation between soluble CD200 concentration and lineage negative MDSC expansion in both our GBM and ependymoma immunotherapy patients as they progressed and went off trial (Figure 1F and Additional file 2: Figure S2 B respectively). These studies demonstrate a direct correlation between sCD200 and the expansion of the suppressive cells. However, we recognize that this study has a low number of patients. Although studies are ongoing to support our observations in humans, we have demonstrated that sCD200 induces expansion of MDSCs in our murine glioblastoma model.

To overcome the immunosuppressive nature of tumors, agents targeting the known immune checkpoints (checkpoint inhibitors) have been developed and are under evaluation as cancer interventions [26-28]. These inhibitors, in combination with current immunotherapy regimens, remarkably enhance the efficacy of anti-tumor immunotherapy. Checkpoint inhibitors block the interactions between ligands and their receptors. Two humanized antibodies, ipilimumab and nivolumab [33], have been developed as checkpoint inhibitors to block negative signals to T cells. In addition, anti-CD200 demonstrated efficacy in a B-cell chronic lymphocytic leukemia [34] that led to the use in a clinical trial (clinicaltrials.gov identifier NCT00648739). Our data demonstrate that CD200R antagonist peptides reverse/block GL261-derived immune suppression within tumor-draining lymph nodes. This reversal is striking given the aggressiveness of GL261 and the many ways it suppresses the immune system. This suggests that CD200 is a central driver of glioma-mediated immune suppression.

## Conclusion

Our current hypothesis is that the CD200R antagonists bind to CD200R on local dendritic cells, protecting them from the suppressive effects of soluble CD200 upon entry into the draining lymph nodes. This hypothesis was determined from preliminary data showing that vaccinating mice with the CD200R antagonist significantly enhanced the expansion of a SIINFEKL/CD8 T cell population (Figure 3A). However, early studies revealed that mice had to be pre-vaccinated with the antagonist prior to tumor lysate vaccination in order to achieve a survival benefit. We speculate that this was due to the presence of sCD200 within our tumor lysates competing for the CD200R on dendritic cells. Ongoing investigations are in progress to verify this. The data presented here are significant and are a part of a more comprehensive study to further understand the effects of CD200 in cancer, specifically how CD200 expands a MDSC population. Mechanistic studies are ongoing and not within the scope of this manuscript. Nevertheless, we demonstrate the potential importance of CD200 in tumor immunotherapy and the use of antagonist peptides as effective agents to block the suppressive effects of sCD200. Our data suggest these antagonist peptides will be useful to enhance immunotherapy not only for glioma, but for breast carcinoma as well. We anticipate that our results will lead to the development of novel inhibitors of the CD200R pathway that can be used as immunotherapy adjuncts to mitigate the suppressive tumor environment in a variety of human cancers.

## Methods

### Patient sample collection

Patients undergoing care for brain tumors were identified and consented prior to surgical biopsy and/or resection. Patients and/or their legal representative were consented according to institutional guidelines. Patients were treated at one of four participating institutions: the University of Minnesota, the University of Pittsburgh, the University of Colorado and UCLA. Patient tissue and blood samples were collected and stored for analysis at the University of Minnesota.

### Western blot analysis

Tissues were minced and sonicated in RIPA lysis buffer containing protease and phosphatase inhibitors (Pierce). Protein concentrations were determined using the bicinchoninic acid colorimetric method (Pierce). Tumor lysates were diluted in reducing sample buffer (Novex) and 50 μg were loaded per lane on a 4% to 12% SDS-PAGE gel (Nu-Page) and run at 160 volts (0.8 volt hours). Gels were then transferred to nitrocellulose at 7 volts (BioRad), blocked using 5% non-fat dry milk/0.05 mM tris buffered saline with 0.05% tween-20 for 1 hr, incubated at 1:1000 in anti-OX2, 200 μg/ml (Santa Cruz) in blocking buffer for 1 hr, and washed six times over 1 hr in TBS/Tween-20. Blots were then incubated at 1:10,000 with anti-goat, 500 μg/0.5 ml IgG HRP (Jackson ImmunoResearch) in blocking buffer for 1 hour and washed six times over 1 hour in TBS/Tween-20. Nitrocellulose was incubated in ECL Plus chemiluminescent substrate (GE) for 1 minute and exposed to HyBlot CL Autoradiography film (Denville Scientific) for 30 seconds.

### Microarray analysis

Data for Figure 1B and D and Additional file 2: Figure S2, transcriptomic microarray profiles of tumor and normal brain tissue samples were generated using Affymetrix HG-U133 Plus 2 GeneChip microarrays (Affymetrix) as previously described [35]. RNA was isolated from specimens using RNeasy or DNA/RNA AllPrep kit (Qiagen) according to manufacturer’s instructions. Tumor specimens from which both RNA and DNA were isolated were determined by histology to contain ≥70% tumor cells and thus had minimal normal tissue contamination. RNA quality was verified using the Nano Assay Protocol for the 2100 Bioanalyzer (Agilent) (RNA integrity number ≥ 8). RNA was amplified, biotin-labeled, and hybridized to Affymetrix HGU133 Plus 2 GeneChips according to manufacturer’s instructions. Analysis of transcriptomic microarray data was performed using Bioconductor functions written in the R programming language (http://www.bioconductor.org). Microarray data CEL files were background corrected and normalized using the guanine cytosine robust Multiarray Average (gcRMA) algorithm, resulting in log2 expression values. Normalized hybridization intensity values for CD200 were obtained from this dataset and averaged for each type and molecular subtype of pediatric brain tumor.

For Figure 1C, total RNA was purified from fresh frozen tumor samples previously collected as part of an IRB-approved research protocol using the RNeasy mini kit (Qiagen). cRNA was generated, quantified and hybridized to U133 Plus 2.0 arrays at the UCLA DNA Microarray Facility using standard Affymetrix protocols. CEL files were normalized using GeneSpring GX 11.5.1 software (Agilent Technologies). To evaluate the baseline expression of CD200 in heterogeneous human brain tumor tissues, we analyzed the relative expression of CD200 using microarray gene expression profiling in over 300 human brain tumor samples and segregated the data by known gene expression signatures. The relative expression of CD200 was normalized and tested. CD200 expression was then plotted together with overall survival using the Probeset Analyzer tool developed at UCLA (http://probesetanalyzer.com).

### Animal models and cell lines

GL261 glioma tumors were implanted into female C57BL/6 mice (6–8 wk old) (Jackson Laboratory) and maintained in a pathogen-free facility according to the guidelines of the University of Minnesota Animal Care and Use Committee. The GL261 orthotopic transplant model was established in C57BL/6 (B6) mice by inoculation with 15,000 GL261-Luc^+^ cells in 1 μl of saline [36]. Tumors were implanted stereotactically into the right striatum; coordinates were 2.5 mm lateral, 0.5 mm anterior of bregma, and 3 mm deep from the cortical surface of the brain [25]. Tumor burden was determined by bioluminescent imaging. Light emitted from the tumors was quantified using Living Image software (Xenogen) and expressed as photons per second per centimeter squared per steradian (p/s/cm^2^/sr). GL261 cells for tumor lysate vaccines were cultured in neural stem cell media consisting of DMEM/F12 (1:1) with L-glutamine, sodium bicarbonate, penicillin/streptomycin (100 U/ml), B27 and N2 supplements (Life Technologies), and 0.1 mg/ml Normocin (Invivogen). Cultures were maintained at 5% O_2_ and supplemented with 20 ng/ml EGF and FGF semiweekly (R&D Systems, Minneapolis, MN). The breast carcinoma EMT6 model was established in BALB/c mice by injection of 1 × 10^6^ EMT6 cells in 50 ml of PBS into the left superior mammary fat pad as described [37]. Mice were euthanized when tumors reached 1000 mm^3^. Mice were challenged with 1 × 10^6^ EMT6 cells in 50 ml of PBS into the left superior mammary fat pad. Tumor cells used to establish both models were cultured in DMEM containing 10% FBS, penicillin/streptomycin (100 U/ml), and 0.1 mg/ml Normocin (Invivogen) in atmospheric oxygen.

### CD200R antagonists

Based on a report from Gorczynski [9], we synthesized the CD200R antagonists 6059 (NTIGDGGCY), 4013 (LFNTFGSQKVSGT) and 4004 (TASLRCSLKTSQE) and for activity (Thermo Fisher). We decided to modify the CD200R antagonist now A26059 (STVHEILCKLSLEGD(dPEG4)NTIGDGGAY) and its irrelevant peptide control was derived by Thermo Scientific (STVHEILCKLSLEGD(dPEG4)LESHLIKVS).

### Vaccine preparation and injection

Tumor lysates were prepared by dissociating tumor cells with non-enzymatic dissociation solution (Sigma). Cells were then washed 3 times with PBS, re-suspended in 500 μL PBS, and frozen overnight at -80°C. Cells were then lysed by five freeze/thaw cycles of freezing in liquid nitrogen and thawing in a 55°C water bath. Cell debris was pelleted by centrifugation at 14,000 RCF, and the protein concentration of the supernatant was determined using a Bradford assay. Lysates were stored at -80°C until use. Each vaccine was prepared on the day of vaccination and consisted of 65 μg of protein tumor lysate and 10 μg Poly:ICLC (a kind gift Dr. Salazar Dr, Oncovir, Inc.), with or without 50 μg of the 6059 peptide antagonist, in a final volume of 100 μL injected intradermally in the back of the neck. For survival studies, vaccines were administered weekly starting three days post-tumor implantation for a total of 6 doses. For T cell priming experiments, animals were vaccinated intradermally on four consecutive days with 100 μg Ovalbumin (OVA) and 10 μg Poly:ICLC, and once more on day eleven [25].

### Flow cytometry

Anti-mouse CD8α-Pacific Blue (clone 53-6.7) was purchased from eBioscience. A SIINFEKL/K^b^ dextramer–PE was used for detection of SIINFEKL/K^b^-binding CD8 T-cells (Immunodex). For whole blood staining, 50 μL of blood was obtained via retro-orbital bleed and placed in 100 μL in heparin and PBS. 5 μL of SIINFEKL/K^b^ dextramer was added to the blood and incubated at room temperature for 10 min. Then 0.5 μg of antibody was added and incubated for an additional 20 min. Red blood cells were lysed by adding 1 mL of 1:10 dilution lysis buffer (BD Pharmigen), incubated for 10 min, washed twice analysis by flow cytometry. Data analysis was performed using FlowJo software. In our human experiments, peripheral blood mononuclear cells and whole blood for serum were isolated from patients at vaccination and on weeks 4, 8, 12, and 24 post vaccination in a recent clinical trial [24]. A 100 μl sample of whole blood was immediately analyzed for percentage of MDSCs ((Lineage-FITC (CD3, CD14, CD16, CD19, CD20, CD56) CD33-APC, HLA-DR-PerCp)) by flow cytometry. Sera CD200 levels were determined by ELISA as instructed by manufacture (Sino Biological Inc, China).

### Cytokine detection

Lymph nodes were homogenized in RPMI media containing 10% FBS, penicillin/streptomycin (100 U/ml), and 0.1 mg/ml Normocin (Invivogen). Cells were filtered using a 70-micron filter, centrifuged and resuspended at a concentration of 500,000 cells/well in triplicate, stimulated with 10 μg of OVA and incubated for 48 hrs. Fifty microliters of supernatant was analyzed for IFN-γ using a flow cytometric bead array according to the manufacturer’s protocol (BD Biosciences).

### MDSC detection and analysis

Spleens were harvested and homogenized in RPMI media containing 10% FBS, penicillin/streptomycin (100 U/ml), and 0.1 mg/ml Normocin (Invivogen). Cells were then filtered using a 70-micron filter, centrifuged, and re-suspended in 5 ml of Ammonium-Chloride-Potassium buffer and incubated for 5 minutes at room temperature to lyse RBCs. Following lysis, cells were washed and plated into 96 well plates at a concentration of 500,000 cells/well in triplicate. Following 24 h incubation, CD200R antagonist peptide A26059 was added to the appropriate wells, incubated for 20 min prior to adding 5 μg of purified CD200 protein (Sino Biological, China) and incubated for a further 72 hrs, harvested and analyzed for anti-CD11b and anti-Ly6c population using an LSR II flow cytometer.

To determine the level of MDSC expansion when splenocytes were exposed to tumor cells, splenocytes were also harvested and purified as described above; 300,000 splenocytes were plated in the bottom of 24 well transwell plates containing a 0.4-micron filter. Following 24 hr incubation, 30,000 GL261 cells were plated on the transwell insert with and without the CD200R antagonist A26059. Total volume was 700 μl/well so that the insert was submerged in the media within the wells. Cells were incubated for 5 days, harvested, and stained with CD11b and GR-1 to determine MDSC levels. In another set of wells, OVA was added to the wells 24 hours after the splenocytes were plated. Following another 72 hr incubation, 3x10^5^ purified CD8 T-cells isolated from an OT-I mouse and were added to the wells and incubated for an additional 72 hrs. Supernatant was harvested and used to determine IFN-γ concentration levels by bead array (BD Biosciences).

### Statistical analysis

Statistical comparisons were made by one-way ANOVA (Kruskal-Wallis test) with post hoc analysis by Dunn’s multiple comparison tests to compare selected groups. Correlations were determined by linear regression with a 95% confidence level plotting the averages of sCD200 concentration in patient’s sera vs. MDSC percentages over time as the patients went off a clinical trial. Differences in animal survival were evaluated by log-rank test. All tests were done with Prism 5.0d software (Graph Pad Software, Inc). *P* values <0.05 were considered significant.

## Additional files

Additional file 1: Figure S1. Variable tissue expression of CD200. (A) mRNA expression levels of CD200 from indicated tissues were analyzed by microarray. (B) mRNA expression levels of CD200 from different regions of normal brains were analyzed by microarray. Means are indicated, statistical significance was determined by one-way ANOVA, post hoc analysis by Dunn’s multiple comparison test, *p < 0.05, **p < 0.001, ***p < 0.0001. Additional file 2: Figure S2. Soluble CD200 concentration increases with tumor reoccurrence. (A) Sera CD200 concentration from an ependymoma patient was monitored overtime throughout a recent clinical trial and (B) compared to patients lineage negative levels as she progressed and went off trial. Means are indicated, statistical significance was determined by one-way ANOVA, post hoc analysis by Dunn’s multiple comparison test, *p < 0.05. R^2^ was determined using linear regression. Additional file 3: Figure S3. Control CD200R antagonist fails to block the suppressive effects of CD200. A. Naïve splenocytes were pulsed with purified CD200 +/- CD200R antagonist or CD200 +/- control antagonist and analyzed for MDSC expansion and arginase-I production. B Tumor bearing mice were vaccinated with saline (n = 5), tumor lysate + CpG (n = 5) or tumor lysate + CpG + antagonist A26059 (n = 5) or tumor lysate + CpG + control antagonist (n = 5). Mice were imaged weekly for tumor growth.
