# Supplementary figures and images for: CD200 in CNS tumor-induced immunosuppression: the role for CD200 pathway blockade in targeted immunotherapy
Source: J Immunother Cancer. 2014 Dec 16;2:46. doi: 10.1186/s40425-014-0046-9 (PMC4296547; doi:10.1186/s40425-014-0046-9)

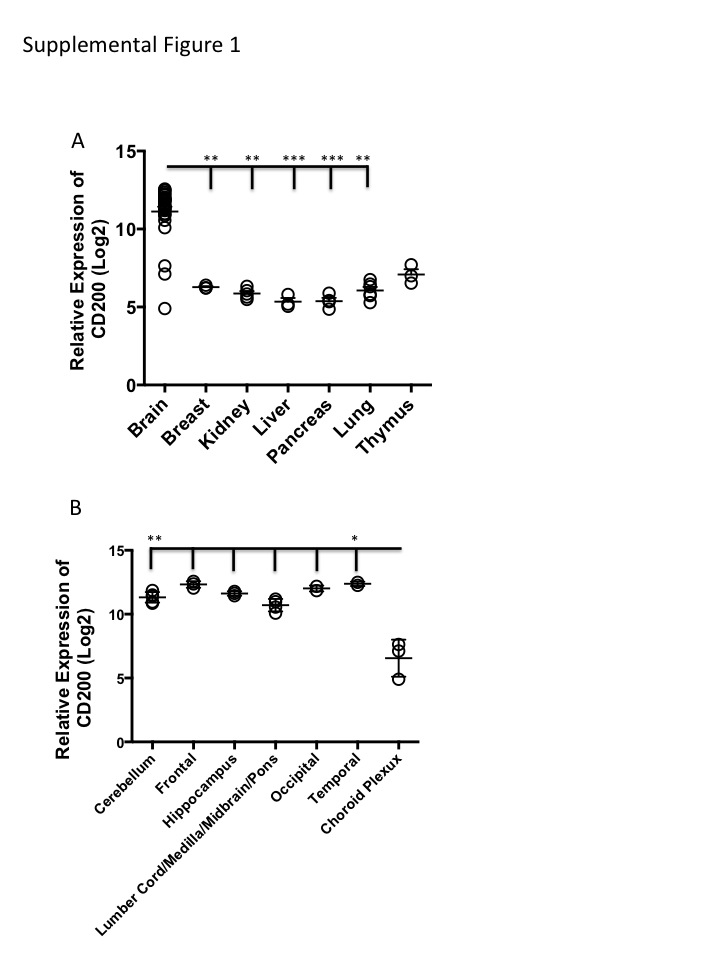

Supplement: Additional file 1: Figure S1. — Variable tissue expression of CD200. (A) mRNA expression levels of CD200 from indicated tissues were analyzed by microarray. (B) mRNA expression levels of CD200 from different regions of normal brains were analyzed by microarray. Means are indicated, statistical significance was determined by one-way ANOVA, post hoc analysis by Dunn’s multiple comparison test, *p < 0.05, **p < 0.001, ***p < 0.0001. [file 40425_2014_46_MOESM1_ESM.jpeg]

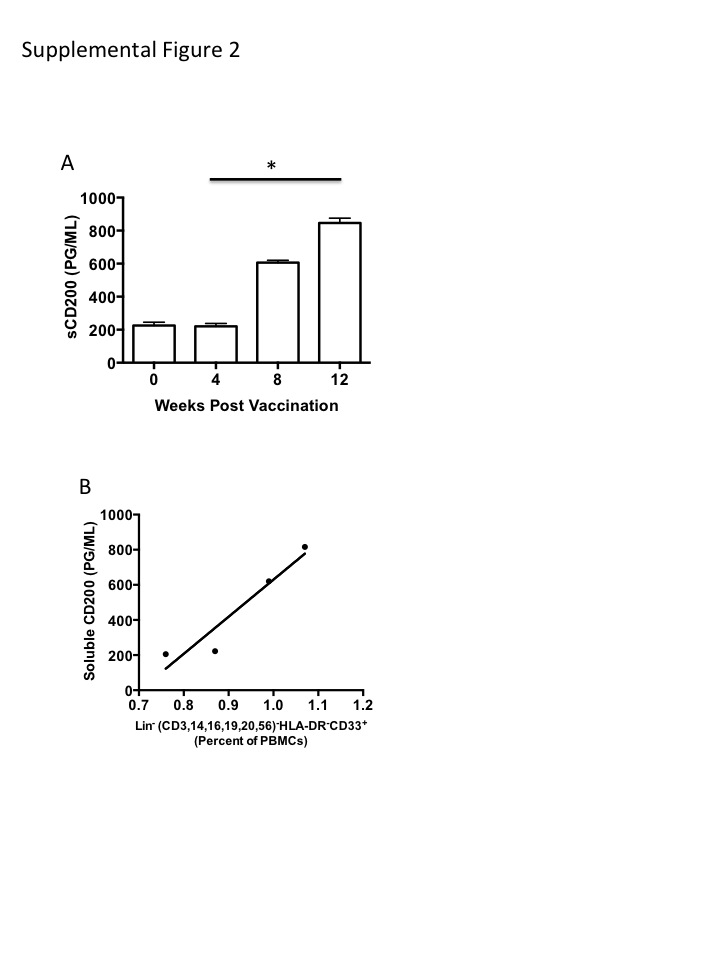

Supplement: Additional file 2: Figure S2. — Soluble CD200 concentration increases with tumor reoccurrence. (A) Sera CD200 concentration from an ependymoma patient was monitored overtime throughout a recent clinical trial and (B) compared to patients lineage negative levels as she progressed and went off trial. Means are indicated, statistical significance was determined by one-way ANOVA, post hoc analysis by Dunn’s multiple comparison test, *p < 0.05. R2 was determined using linear regression. [file 40425_2014_46_MOESM2_ESM.jpeg]

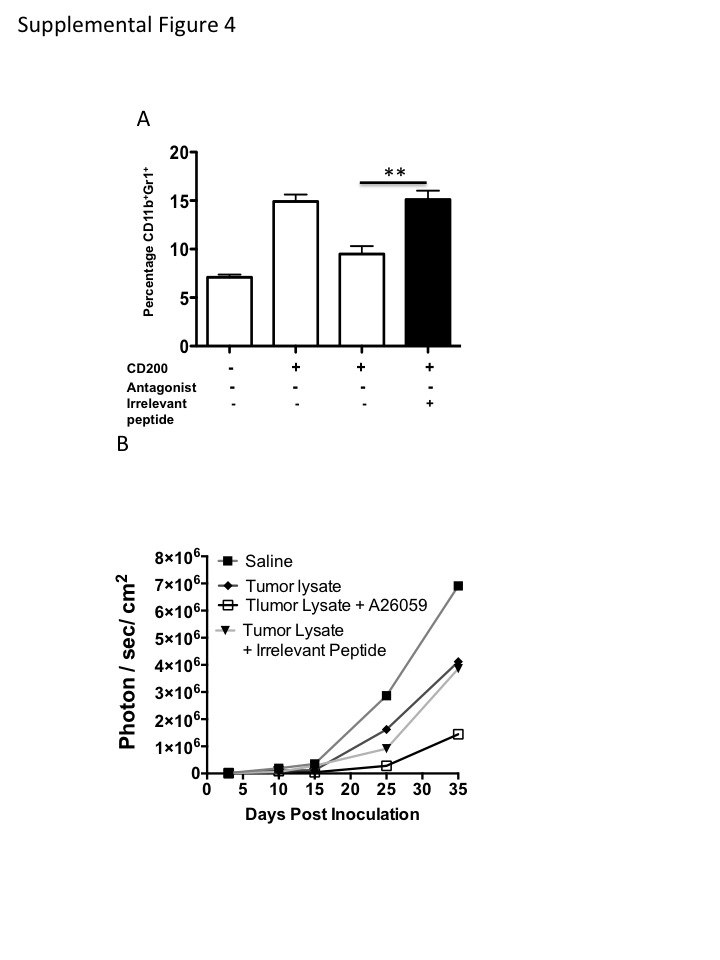

Supplement: Additional file 3: Figure S3. — Control CD200R antagonist fails to block the suppressive effects of CD200. A. Naïve splenocytes were pulsed with purified CD200 +/- CD200R antagonist or CD200 +/- control antagonist and analyzed for MDSC expansion and arginase-I production. B Tumor bearing mice were vaccinated with saline (n = 5), tumor lysate + CpG (n = 5) or tumor lysate + CpG + antagonist A26059 (n = 5) or tumor lysate + CpG + control antagonist (n = 5). Mice were imaged weekly for tumor growth. [file 40425_2014_46_MOESM3_ESM.jpeg]
